# Seven Years of *Salmonella*: Changing Resistance and Clinical Insights

**DOI:** 10.3390/microorganisms13122655

**Published:** 2025-11-22

**Authors:** Cristina Mihaela Sima, Aida Corina Bădescu, Georgiana Buruiană, Alexandru Duhaniuc, Luminița Smaranda Iancu, Eduard-Vasile Năstase, Olivia Simona Dorneanu

**Affiliations:** 1 Grigore T. Popa University of Medicine and Pharmacy Iasi, 700115 Iași, Romania; cristina.sima@umfiasi.ro (C.M.S.); aida.badescu@umfiasi.ro (A.C.B.); georgiana.buruiana@umfiasi.ro (G.B.); alexandru.duhaniuc@umfiasi.ro (A.D.); eduard-vasile.nastase@umfiasi.ro (E.-V.N.); olivia.dorneanu@umfiasi.ro (O.S.D.); 2 Clinical Hospital of Infectious Diseases “Sfânta Parascheva”, 700116 Iași, Romania; 3 Iași Regional Center for Public Health, National Institute of Public Health, 700465 Iași, Romania

**Keywords:** foodborne pathogens, invasive NTS, Eastern Europe, fluoroquinolones, hospitalization

## Abstract

Non-typhoidal *Salmonella* (NTS) represents a growing public health concern worldwide due to its increasing antimicrobial resistance and substantial disease burden, yet data from Romania remain limited. We conducted the first regional 7-year retrospective study of NTS-associated diarrhea among hospitalized patients, focusing on resistance trends and clinical factors associated with disease severity. This study included all laboratory-confirmed *Salmonella* infections admitted for acute diarrheal disease to the “Sfânta Parascheva” Clinical Hospital of Infectious Diseases, Iași (January 2018–December 2024). Patient data were extracted from electronic medical records and analyzed using SPSS (v31.0), with statistical significance set at *p* < 0.05. Among the isolates obtained from the 698 included patients, most belonged to serogroup D (63.6%), followed by B (21.8%) and C (14.0%). Overall resistance rates were 12.3% for ampicillin, 3.6% for trimethoprim–sulfamethoxazole and 29.9% for ciprofloxacin, with a significant yearly increase observed only for ciprofloxacin (OR = 1.21, *p* < 0.001). Cardiovascular comorbidities were independently associated with prolonged hospitalization (>5 days) (OR = 2.25, *p* = 0.007). Invasive infections occurred in 14 patients (2%). Given the high ciprofloxacin resistance and the additional impact of comorbidities on disease severity, there is a need for ongoing surveillance and targeted management strategies.

## 1. Introduction

In recent years, NTS has emerged as a significant public health concern, primarily due to its rising incidence, foodborne transmission and substantial burden on morbidity and mortality, leading to increased healthcare costs [1,2,3,4]. Each year, NTS accounts for approximately 150 million cases of illness and 60,000 deaths globally [5]. In 2022, European countries reported 66,721 cases of salmonellosis, with 65,967 laboratory-confirmed and 39.3% of those with known status requiring hospitalization [6]. Within the region, *Salmonella* ranked as the second most frequently reported foodborne pathogen after *Campylobacter* [7]. Nonetheless, the true burden of disease is likely underestimated, as for every reported case, approximately 57 additional infections remain undetected [8].

NTS typically causes mild gastroenteritis, characterized by diarrhea, vomiting and abdominal pain [9]. Transmission occurs through person-to-person contact or from zoonotic reservoirs, including poultry, swine, cattle and wild animals [10]. Most infections are associated with contaminated food or water [11], with an estimated 94% attributable to foodborne transmission [12]. Although NTS infections typically present as self-limiting gastroenteritis, they may progress to invasive disease (iNTS) in vulnerable populations, resulting in bacteremia or meningitis [13,14,15]. Recent global epidemiological reports indicate that iNTS infections are increasingly recognized as a distinct clinical and public health concern, particularly due to their association with multidrug resistance (MDR) phenotypes and severe outcomes [14,16]. The burden of invasive disease is highest in sub-Saharan Africa, where case-fatality rates can exceed 20% [17,18,19]. Beyond acute illness, chronic *Salmonella* infections have also been linked to long-term sequelae, including an increased risk of colorectal cancer [20,21]. According to the White-Kauffmann-Le Minor classification scheme, more than 2500 *Salmonella* serovars have been identified [22]. The most frequent serovars implicated in foodborne outbreaks are *S.* Heidelberg [23,24], *S.* Typhimurium [25] and *S.* Enteritidis, which remains one of the most prevalent serovars associated with foodborne transmission [26].

The global rise in antimicrobial resistance among NTS is a major public health concern, as resistant strains are associated with increased risks of bloodstream invasion and hospitalization [27]. NTS has developed resistance to key antibiotics, with recent reports indicating resistance rates up to 30% for ciprofloxacin [28] and 50.8–73.4% for ampicillin [29,30]. Resistance to sulfonamides is also common, observed in approximately 43% of isolates [28]. Multidrug resistance (MDR) rates are particularly high, reaching nearly 50% in sub-Saharan Africa [31,32].

Although surveillance data from Romania on human NTS infections are limited, recent reports from animal sources show alarming MDR rates, over 90% in pork-derived isolates [33] and increasing rates in poultry isolates [34], highlighting the need for updated human-focused studies. The lack of longitudinal human clinical data creates a gap in understanding the local epidemiology, resistance dynamics and risk factors associated with severe or invasive disease. Given the recent reports demonstrating high MDR rates in animal reservoirs in our region, together with evidence that MDR strains have an increased likelihood of progressing to invasive disease, updated human-focused surveillance is essential. To address this gap, we conducted a 7-year retrospective study of NTS infections in northeastern Romania, representing the first study from this region to evaluate antimicrobial resistance trends and clinical determinants of disease severity.

## 2. Materials and Methods

### 2.1. Study Design and Data Collection

This was a single-center, retrospective study conducted to characterize NTS infections among hospitalized patients in northeastern Romania. This study included all patients with a laboratory-confirmed *Salmonella* infection identified by stool culture, who were admitted with acute diarrheal disease at “Sfânta Parascheva” Clinical Hospital of Infectious Diseases in Iași, between 1 January 2018 and 31 December 2024. Patients with a negative stool culture or a positive culture for pathogens other than *Salmonella* were excluded. Stool samples were inoculated onto Hektoen Enteric Agar (HEA; Oxoid, Basingstoke, UK) and Brilliance *Salmonella* Agar (BSA; Oxoid, Basingstoke, UK) and incubated at 37 °C for 16–24 h. Colonies with typical morphology (lactose negative/black centers on HEA, purple on BSA) were selected for biochemical testing using Triple Sugar Iron Agar (TSI; Oxoid, Basingstoke, UK), Sulfide Indole Motility medium (SIM; Oxoid, Basingstoke, UK) and Urea Agar Base (Oxoid, Basingstoke, UK). Presumptive *Salmonella* isolates were confirmed by slide agglutination with *Salmonella* polyvalent OMA and OMB antisera (Sifin Diagnostics GmbH, Berlin, Germany). Confirmed isolates were subcultured on Drigalski Lactose Agar (DLA; Oxoid, Basingstoke, UK) for subsequent serogrouping and antimicrobial susceptibility testing. Serogroup identification was performed by slide agglutination with *Salmonella* polyvalent and monovalent antisera O:3,10, O:4,5, O:6, O:7, O:8, O:9 and O:13 (Sifin Diagnostics GmbH, Berlin, Germany). Antimicrobial susceptibility testing was conducted using the disk diffusion method, including ampicillin (AMP), trimethoprim-sulfamethoxazole (SXT) and ciprofloxacin (CIP) (with pefloxacin 5 μg used as the screening agent for CIP resistance). The susceptibility results were interpreted according to the European Committee on Antimicrobial Susceptibility Testing (EUCAST) guidelines valid at the time of testing.

Patient data were extracted from electronic medical records and included demographics (age, sex, residence), duration of hospitalization and the presence of systemic comorbidities such as diabetes, cardiovascular disease, pulmonary disease or malignancy (all types). Laboratory values at admission were also collected, including white blood cell (WBC), neutrophil and red blood cell (RBC) counts, hemoglobin, C-reactive protein (CRP), and electrolytes levels. These parameters were collected because they represent routinely used indicators of systemic inflammation, infection severity and hydration status in acute bacterial gastroenteritis. Invasive *Salmonella* infections were identified based on positive cultures from non-stool samples (like blood or urine) to differentiate systemic from localized disease.

### 2.2. Statistical Analysis

All statistical analyses were performed using IBM SPSS Statistics, version 31.0.0.0 (117) and a *p*-value of <0.05 was considered statistically significant.

Descriptive statistics summarized clinical, demographic and antimicrobial resistance data. Continuous variables were reported as mean ± SD for normally distributed data or as median (IQR: Q1–Q3) for non-normally distributed data; categorical variables as counts and percentages. For resistance rates, 95% confidence intervals were calculated using the Wilson method.

To assess whether *Salmonella* serogroups were associated with differences in clinical presentation, admission laboratory values were compared using ANOVA test for data with a normal distribution and Kruskal–Wallis test for data with a non-normal distribution. The association between *Salmonella* serogroups and resistance to each antibiotic was initially evaluated using Pearson’s Chi-square tests. Yearly variations were described by examining annual distributions of serogroups and resistance rates. To explore potential changes in resistance patterns over time, separate binary logistic regression models were fitted for each antibiotic, with resistance coded as resistant (R) versus susceptible (S) and year entered as an ordinal predictor. For CIP, an additional model adjusted for *Salmonella* serogroup was constructed.

Risk factors for prolonged hospitalization were evaluated in relation to clinical, demographic, biological and antimicrobial resistance data. As hospitalization duration was not normally distributed, non-parametric tests were applied: Spearman’s correlation for continuous variables, Mann–Whitney U test for comorbidity and resistance comparisons and Kruskal–Wallis test for differences across serogroups. To identify independent predictors of prolonged hospitalization, a binary logistic regression model was constructed. Prolonged hospitalization was defined as a stay exceeding 5 days, as this threshold corresponded to the median length of stay in the cohort and provided a clinically meaningful way to stratify patients. Variables that showed significant associations in univariate analyses were entered into the model.

Risk factors for invasive disease were evaluated in univariate analyses, comparing demographic, clinical and laboratory variables between invasive and non-invasive cases. Given the small number of invasive infections, continuous variables were analyzed using the Mann–Whitney U test, while categorical variables were assessed with Fisher’s exact test. Due to the limited sample size (14 invasive cases), no multivariable analysis was conducted.

### 2.3. Ethics Statement

This study was conducted in full accordance with international medical ethics standards, as outlined in the Declaration of Helsinki. This study was approved by the Ethics Committee of “Sfânta Parascheva” Clinical Hospital of Infectious Diseases, Iași, Romania and by the University Ethics Committee of “Grigore T. Popa” University of Medicine and Pharmacy, Iași, Romania. The requirement for written informed consent was waived by Ethics Committee of “Sfânta Parascheva” Clinical Hospital of Infectious Diseases, Iași, as this study used fully anonymized patient data from existing electronic medical records.

## 3. Results

**Figure 1:**
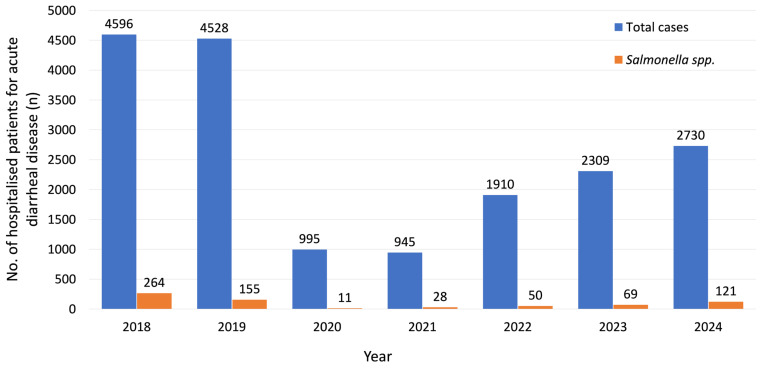
Annual distribution of hospitalized cases of acute diarrheal disease and *Salmonella*-associated diarrhea (2018–2024).

Between 2018 and 2024, a total of 18,013 individuals were hospitalized due to acute diarrheal disease, of which 698 cases were attributed to *Salmonella* infection. The number of cases varied annually, as illustrated in Figure 1.

### 3.1. Baseline Profile of the Study Population

A total of 698 patients hospitalized with *Salmonella* diarrheal disease were identified from electronic medical records between 2018 and 2024 and included in this study. To characterize the study population, descriptive demographic and clinical characteristics (including age distribution, sex, residence, hospitalization duration and comorbidities) are presented in Table 1. The cohort showed a slight predominance of male patients and a higher proportion of individuals from rural areas. Most cases occurred in adults, with a median hospitalization duration of 5 days (IQR: 3–6). The most frequent comorbidities involved cardiovascular and metabolic disorders. Year-to-year variations in all analyzed parameters are presented in Supplementary Materials Table S1.

Most biological parameters measured at admission showed non-normal distributions, as indicated by significant the Shapiro–Wilk test results (*p* < 0.001), except for hemoglobin (*p* = 0.312) and serum potassium (*p* = 0.187). These variables were summarized using median and interquartile range (IQR), while those with normal distributions were expressed as mean ± standard deviation (SD). Table 2 presents the results descriptively for the overall study population and stratified by serogroup, with normal ranges provided for clinical interpretation.

### 3.2. Characterization of Circulating Salmonella Serogroups

Next, we assessed the distribution of *Salmonella* serogroups isolated from hospitalized patients during the study period. Serogroup D emerged as the most prevalent, accounting for 63.6% of all isolates, followed by serogroups B (21.8%) and C (14%). Other serogroups, including E, G, and unknown (UN), were infrequently detected, each constituting less than 1% of the total isolates. Specifically, two isolates belonged to serogroup E, one to serogroup G and one *Salmonella* isolate lacked serogroup information in the database. Due to the limited number of these rare serogroups, they were excluded from further analysis to minimize the risk of statistical bias associated with low expected counts. A detailed evaluation of the serogroup distribution across the years is presented in Table 3.

To explore associations between *Salmonella* serogroups and laboratory profiles at admission, normally distributed variables were compared using the ANOVA test and non-normally distributed variables using the Kruskal–Wallis test. Significant differences across serogroups were observed for WBC count, serum sodium, serum chloride and hemoglobin levels (all *p* < 0.05). Post hoc analyses indicated that values for these parameters differed significantly for serogroup D compared with one or both of the other serogroups. No significant differences were found for the remaining parameters (all *p* > 0.05).

**Figure 2:**
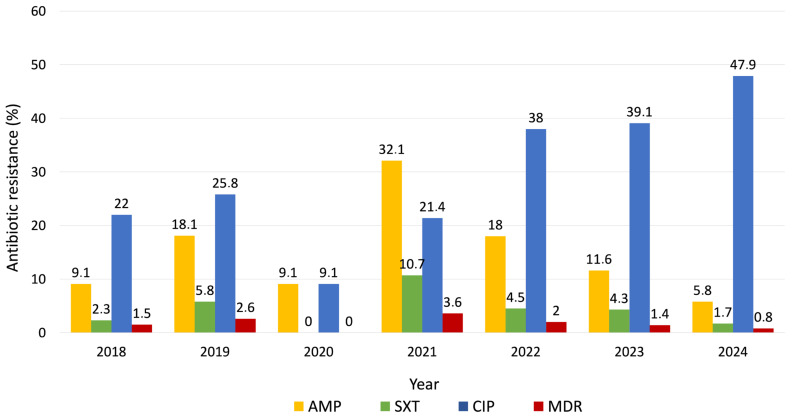
Trends of antibiotic resistance during the study period (2018–2024).

Antibiotic susceptibility testing conducted during the study period revealed that 12.3% (95% CI 10.1–15.0%) of isolates were resistant to AMP, while 3.6% (95% CI 2.5–5.3%) showed resistance to SXT. Notably, CIP resistance was higher, detected in 29.9% (95% CI 26.7–33.4%) of isolates. MDR, defined as resistance to at least three different classes of antibiotics, was identified in 1.7% of all cases. Resistance to AMP fluctuated over time, with a transient peak in 2021. SXT resistance remained consistently low throughout the study period, while CIP resistance showed a progressive increase, nearly doubling by 2024. Yearly variation in antimicrobial resistance rates was illustrated in Figure 2.

**Figure 3:**
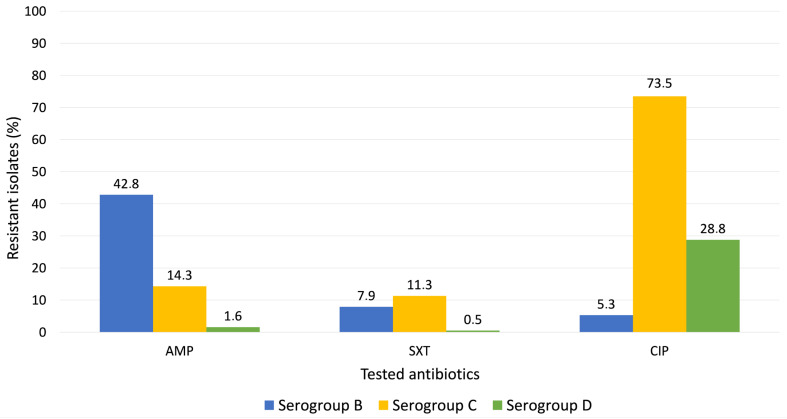
Distribution of antimicrobial resistance by *Salmonella* serogroup.

To explore whether yearly variations in resistance rates were influenced by the distribution of circulating *Salmonella* serogroups, Chi-square tests were performed to assess the association between serogroup and resistance to each antibiotic. Statistically significant associations were observed for all tested antimicrobials: AMP (χ^2^(2, N = 694) = 177.31, *p* < 0.001), SXT (χ^2^(2, N = 688) = 37.17, *p* < 0.001) and CIP (χ^2^(2, N = 694) = 132.83, *p* < 0.001). Resistance rates across serogroups are illustrated in Figure 3, showing notably higher AMP and SXT resistance in serogroups B and C, while CIP resistance predominated in serogroup C.

Since significant differences in resistance rates were observed among serogroups, we next assessed whether overall resistance patterns changed over time by applying binary logistic regression models for each antibiotic. A statistically significant temporal increase was observed only for CIP resistance (OR = 1.21; 95% CI 1.13–1.29; *p* < 0.001), while no significant temporal changes were detected for AMP or SXT. In the multivariable model including year and serogroup, both variables remained significant (*p* < 0.001) and the effect of year persisted after adjustment (OR = 1.24; 95% CI 1.15–1.33).

### 3.3. Risk Factors for Prolonged Hospitalization

To explore possible risk factors for prolonged hospitalization, associations were assessed between the length of hospital stay and admission parameters, age, comorbidities and antimicrobial resistance. Spearman’s correlation showed significant positive correlation with age (ρ = 0.192, *p* < 0.001) and negative correlations with RBC (ρ = –0.169), hemoglobin (ρ = –0.134) and serum potassium (ρ = –0.162), all *p* < 0.001. No other parameters were significantly correlated. The Mann–Whitney U test showed significantly longer hospital stays in patients with diabetes and cardiovascular disease (*p* < 0.001). Pulmonary and oncologic comorbidities had no significant effect (*p* = 0.191 and *p* = 0.096), though cancer showed a trend toward longer stays (mean ranks 435.14 vs. 345.70). No significant differences in hospitalization duration were found based on antibiotic resistance (AMP: *p* = 0.329; CIP: *p* = 0.215; SXT: *p* = 0.886).

Variables that showed significant associations with hospitalization duration in univariate analyses were subsequently included in a multivariable binary logistic regression model to identify independent predictors of prolonged stay. Hospital stay was dichotomized (≤5 vs. >5 days) for clinical relevance. The model was statistically significant (χ^2^(6) = 53.308, *p* < 0.001) and correctly classified 67.2% of cases. Cardiovascular comorbidities were a significant independent predictor (OR = 2.25, 95% CI: 1.25–4.08, *p* = 0.007). Diabetes showed a trend toward significance (OR = 2.11, 95% CI: 0.93–4.85, *p* = 0.076), while age, hemoglobin, RBC and serum potassium were not significant.

### 3.4. Invasive Salmonella Infections

A total of 14 invasive *Salmonella* infections were identified, representing approximately 2% of all cases in the dataset. Patients were predominantly older (median age 71.5 years) and required prolonged hospitalization (median 12 days). Cardiovascular disease was the most frequent comorbidity (42.9%) and serogroup D accounted for the majority of isolates (64.3%). CIP resistance was observed in 35.7% of cases, while no MDR was detected. Detailed demographics, clinical characteristics and admission laboratory parameters are presented in Supplementary Materials Table S2.

To explore factors potentially associated with invasive disease, univariate analyses were performed comparing demographic, clinical and laboratory variables between invasive and non-invasive cases. Older age was associated with invasive infections, as shown by the Mann–Whitney U test (*p* < 0.001). No significant differences were found between invasive and non-invasive groups in WBC, CRP, electrolytes or hemoglobin levels (*p* > 0.05). Fisher’s exact test revealed significant associations between invasive infection and diabetes (*p* = 0.041), cardiovascular disease (*p* = 0.018) and malignancy (*p* = 0.034), but not pulmonary disease (*p* = 0.351). CIP resistance was not significantly linked to invasive infections (*p* = 0.769).

## 4. Discussion

The key findings of our study, based on data collected between 2018 and 2024, highlight several important epidemiological and clinical patterns in *Salmonella* diarrheal disease. A higher number of cases were recorded in 2018 and 2019, with a sharp decline in 2020 and 2021, likely influenced by the COVID-19 pandemic. Reduced healthcare-seeking behavior, changes in food consumption, improved personal protective measures (such as frequent handwashing and mask use), as well as shifts in surveillance priorities, may have contributed to underreporting of *Salmonella* infections, potentially limiting the representativeness of the data for 2020 and 2021. A similar trend was reported in Europe, with the European Food Safety Authority (EFSA) and the European Centre for Disease Prevention and Control (ECDC) noting a 19.6% decline in salmonellosis notification rates in 2020–2021 compared with 2017–2019 [7]. Most patients were from rural areas (58.6%), which may indicate differences in exposure risks, food handling practices or healthcare access compared to urban populations. The predominance of rural cases observed in our cohort is consistent with patterns reported in other studies [35,36]. The length of hospitalization remained stable across the years (median = 5 days), indicating consistency in clinical management and was shorter than what has been reported in other studies [37,38].

In our laboratory, identification of *Salmonella* isolates was restricted to the serogroup level. The lack of serotype-level identification reflects available diagnostic capacity rather than epidemiological intent, leading to reduced comparability with European surveillance data, where serotype-specific reporting is standard. Consequently, interpretation of trends and strain-level dynamics must be made with caution. *Salmonella* serogroup D was predominant in our region (63.6%). This distribution closely mirrors European patterns, where *Salmonella* Enteritidis, a serogroup D strain, remains the most prevalent serotype [39,40], primarily associated with imported poultry [41,42] and eggs [43]. Although not yet predominant in our setting, serogroup B *Salmonella* warrants close monitoring as *Salmonella* Infantis, a serogroup B strain, has increasingly been reported in food sources in the United Kingdom [41,42] and shows high levels of antimicrobial resistance [44,45].

The analysis of baseline laboratory parameters at admission showed that most patients presented with values within normal ranges, suggesting that *Salmonella* diarrheal disease in our cohort generally did not induce major hematological or electrolyte disturbances. However, CRP levels were markedly elevated, with a median of 84.7 mg/L, indicating a significant acute-phase inflammatory response. This observation aligns with previous reports showing that CRP levels are elevated irrespective of bacteremia [46,47]. The systemic CRP increase likely results from mucosal invasion by *Salmonella*, which triggers local cytokine release, particularly IL-6, stimulating hepatic CRP synthesis [48]. Additionally, lipopolysaccharide in the *Salmonella* outer membrane binds to TLR4/CD14, initiating a robust pro-inflammatory cytokine cascade that further amplifies CRP production [49].

We next assessed whether distinct *Salmonella* serogroups were associated with differences in routine laboratory parameters at admission, aiming to identify potential serogroup-related clinical variations. Although statistically significant differences were observed for WBC, serum sodium, serum chloride and hemoglobin levels, these variations were small, suggesting limited clinical relevance. However, since in our study strain identification was limited to the serogroup level, potential serotype-specific effects may have been masked. This is particularly relevant, as prior evidence has shown that specific serotypes may influence disease severity and clinical outcomes [50].

Antimicrobial resistance patterns were further analyzed. In our laboratory, routine antimicrobial susceptibility testing for *Salmonella* included only three agents: AMP, SXT and CIP. While this reflects local diagnostic practices and empirical treatment choices, it limits direct comparison with broader European datasets. Overall, the circulating *Salmonella* strains in our region remained largely susceptible to the tested antibiotics. Resistance to AMP was detected in 12.3% of isolates, declining to 5.8% in 2024. This level was considerably lower than the 25.2% reported across Europe [6] and closer to the 6.6% observed in the United States [51]. A similar pattern was found for SXT, with an overall resistance rate of 3.6% and a temporary peak of 10.7% in 2021, markedly below the 25.6% reported in European data [6]. In contrast, resistance to CIP was notably higher (29.9%), rising to 47.9% in 2024, exceeding the reported levels in Europe (14.9%) [6], the United States (3%) [51] and China (16.2%) [30]. This finding is consistent with recent Romanian data: a 2025 study conducted in the North-East region reported that 63.2% of *Salmonella* strains isolated from diarrheal cases were resistant to CIP [52], supporting the high local prevalence of fluoroquinolone resistance. The proportion MDR isolates was low, accounting for only 1.7% of all cases, substantially below the rates reported in Europe (22.6%) [6], the United States (10.3%) [30], and China (41–80%) [28,53]. However, the low MDR percentage observed in our study should be interpreted with caution, as MDR estimation in our cohort was limited by the restricted antibiotic panel tested, which likely underrepresents the true burden of MDR. Ongoing surveillance of MDR bacteria is crucial, as infections with resistant strains are associated with poorer clinical outcomes, longer hospital stays, extended antimicrobial treatment courses and substantially higher healthcare costs [54,55].

To better understand the factors underlying annual variations in antimicrobial resistance, we examined the relationship between the temporal distribution of *Salmonella* serogroups and resistance rates for each antibiotic. The distribution of serogroups over time appeared to influence the evolution of resistance patterns. For instance, the decline in AMP resistance from 18.1% in 2019 to 5.8% in 2024 paralleled the increasing proportion of serogroup D, which exhibited the lowest resistance rate to AMP (1.6%) compared with serogroup B (42.8%) and C (14.3%). A similar pattern was observed for SXT, where resistance decreased to 1.7% in 2024, coinciding with the predominance of serogroup D (0.5% resistance). Conversely, CIP resistance followed an opposite trajectory, rising from 22% in 2018 to 47.9% in 2024. The increasing trend in CIP resistance observed in our study was further confirmed by the binary logistic regression model, indicating a 21% (OR = 1.21) relative increase in the odds of resistance per year over the study period. Importantly, this association remained significant even after adjusting the model for *Salmonella* serogroup (*p* < 0.001), suggesting that the upward trend in fluoroquinolone resistance was not solely driven by changes in serogroup distribution. In Romania, extensive antibiotic use in livestock may contribute to this trend. A 2011 food survey reported CIP resistance in 42.9% of *Salmonella* isolates from meat sources [56] and a 2024 study found MDR in 93.8% of pork-derived *Salmonella* isolates [33]. Beyond agricultural antimicrobial exposure, Romania’s high community antibiotic consumption and insufficient stewardship infrastructure may be contributing factors to the high fluoroquinolone resistance rates [57].

In order to identify patients at risk of prolonged hospitalization, we analyzed demographic, clinical and admission laboratory parameters that could be associated with longer hospital stays. Significant correlations were observed between length of stay and age, RBC count, hemoglobin and serum potassium. These findings are consistent with previous reports indicating longer hospitalizations among older patients [58] and those with anemia [59]. However, these effect sizes were small, suggesting that the observed associations, while statistically significant, may have limited clinical relevance. Prolonged stays were also associated with diabetes and cardiovascular disease, in line with prior studies [58]. In contrast to earlier evidence [37], antimicrobial resistance was not significantly related to hospitalization duration. This discrepancy may reflect uneven group sizes between resistant and susceptible isolates, timely administration of effective alternative therapies or population differences across studies. To further identify independent predictors of prolonged hospitalization, a binary logistic regression was performed using a clinically meaningful threshold of >5 days, corresponding to the median hospital stay. Cardiovascular disease emerged as an independent predictor, consistent with the univariate analysis, with affected patients being more than twice as likely to experience hospital stays longer than 5 days (OR = 2.25). Although diabetes did not reach statistical significance (*p* = 0.076), the observed odds ratio (OR = 2.11) indicates a possible independent effect that may hold clinical relevance. Conversely, age, hemoglobin, RBC count and serum potassium, previously associated with hospitalization length in univariate analysis, were not retained as significant in the multivariable model, suggesting that their effects may be mediated by comorbid status. These results corroborate earlier evidence showing that cardiovascular and metabolic comorbidities independently contribute to prolonged recovery and hospitalization in *Salmonella* infections [60,61].

Invasive *Salmonella* infections accounted for 2% of all cases in our cohort, a proportion consistent with previous reports, highlighting their relative rarity in non-typhoidal salmonellosis [4,62,63]. Serogroup D predominated (64.3%), in agreement with evidence that *Salmonella* Enteritidis (a serogroup D strain) is frequently linked to invasive disease [60]. CIP resistance was detected in 35.7% of invasive isolates, substantially higher than resistance rates reported in most international studies [64,65,66], though comparable to certain regional data [27]. This finding raises concern regarding the effectiveness of fluoroquinolones in treating severe infections in our setting. To identify risk factors for invasive disease, we compared demographic, clinical and admission laboratory parameters between invasive and non-invasive cases. Invasive infections were significantly associated with older age. Advanced age is known to contribute to immunosenescence, accumulation of comorbidities and delayed pathogen clearance, which together may facilitate systemic dissemination of *Salmonella* [67]. Cardiovascular disease, diabetes mellitus and malignancy were also significantly associated with invasive disease, consistent with previous research [68,69,70,71]. Interestingly, no significant differences were found in admission inflammatory or electrolyte parameters between invasive and non-invasive cases, suggesting that routine laboratory markers are poor predictors of invasive disease at presentation. CIP resistance likewise showed no significant association with invasiveness, despite previous studies reporting a positive link between fluoroquinolone resistance and systemic spread [72]. One possible explanation is that local *Salmonella* strains may carry resistance determinants that are not genetically linked to virulence plasmids, as observed in other geographical settings [73,74]. Also, the relatively small number of invasive cases may have limited the statistical power to detect subtle associations between resistance and invasiveness.

This study had several limitations that should be acknowledged. First, its retrospective design inherently limited control over data quality and completeness, as information was extracted from existing medical records. Second, the single-center nature of this study may constrain the generalizability of the findings to other populations or healthcare settings. Among the 698 hospitalized patients, only 14 had invasive *Salmonella* infections, which limits the statistical power to detect reliable associations or predictors of invasiveness. Therefore, the findings related to invasive disease should be interpreted as exploratory signals rather than definitive associations. Laboratory testing was restricted to serogroup identification without further serotyping, reflecting the diagnostic capacity routinely available in our institution during the study period. This limitation is common in hospital-based laboratories across the region, where routine workflows prioritize rapid identification and basic susceptibility testing to support clinical decision-making. However, the lack of serotype-level characterization reduces epidemiological granularity and limits comparability with European surveillance data, where NTS is routinely reported at the serotype level. Antimicrobial susceptibility testing included only three agents (AMP, SXT and CIP), consistent with routine diagnostic practices and empirical treatment protocols in our setting. Although the testing panel was limited in scope, it remains clinically relevant and adequate for guiding therapeutic decisions for NTS in routine clinical practice.

## 5. Conclusions

This retrospective study, conducted at the “Sfânta Parascheva” Clinical Hospital of Infectious Diseases in Iași, Romania, underscores the ongoing clinical relevance of *Salmonella* as a cause of acute diarrheal disease among hospitalized patients. The predominance of serogroup D and the high rate of CIP resistance emphasize the need for sustained local antimicrobial resistance monitoring and strengthened antibiotic stewardship programs tailored to regional trends.

Beyond microbiological findings, our results highlight the importance of host-related factors in determining disease course and outcomes. Cardiovascular disease emerged as an independent predictor of hospitalization lasting more than 5 days, while diabetes showed a potential contributory role. These observations reinforce the need for proactive risk stratification and closer clinical monitoring in patients with chronic comorbidities.

The small number of invasive cases in our cohort limits the ability to draw firm conclusions. However, the observation that iNTS predominantly occurred in older patients with multiple comorbidities rather than in association with distinct laboratory abnormalities suggests that host vulnerability may play a more important role in progression to invasive disease than routinely measured biomarkers. These findings should be considered exploratory and larger studies are needed to confirm this pattern. Nonetheless, they highlight the potential value of developing clinical risk stratification tools to support earlier recognition and targeted management of patients at increased risk for invasive infection.

Future prospective, multicenter studies integrating detailed serotyping, expanded antimicrobial panels and molecular characterization are warranted to better understand resistance mechanisms and epidemiological patterns. Such efforts will refine both local surveillance and empirical treatment strategies, ultimately enhancing patient outcomes and guiding public health responses to *Salmonella* infections.

## Figures and Tables

**Table 1:** Demographic and clinical characteristics of patients hospitalized with *Salmonella* diarrheal disease (2018–2024).

Parameter	2018–2024
Number of patients (n)	698
Sex (n, %)	
Male	367 (52.6%)
Female	331 (47.4%)
Residence (n, %)	
Urban	289 (41.4%)
Rural	409 (58.6%)
Age (years)	
Median (IQR: Q1–Q3)	25 (IQR: 10–50.25)
<18 years (n, %)	252 (36.1%)
≥18 years (n, %)	446 (63.9%)
Length of hospital stay (days)	
Median (IQR: Q1–Q3)	5 (IQR: 3–6)
Comorbidities (n, %)	
Diabetes mellitus	40 (5.7%)
Cardiovascular disease	117 (16.8%)
Pulmonary disease	21 (3%)
Malignancy (all types)	15 (2.1%)

**Table 2:** Descriptive laboratory parameters in hospitalized NTS cases, reported overall and stratified by serogroup.

Parameter	All Infections	Serogroup B	Serogroup C	Serogroup D	Normal Range
WBC * (cells/mm^3^)					4000–10,000
Median (IQR: Q1–Q3)	7750 (IQR: 5855–10,015)	8175 (IQR: 6302.5–11,075)	7969 (IQR: 5810–9715)	7335 (IQR: 5720–9795)	
Neutrophils (cells/mm^3^)					2000–8000
Median (IQR: Q1–Q3)	5010 (IQR: 3480–7140)	5180 (IQR: 3487.5–7150)	5520 (IQR: 3525–6815)	4970 (IQR: 3497.5–7242.5)	
RBC * (cells/mm^3^)					4,400,000–5,800,000
Median (IQR: Q1–Q3)	4,590,000 (IQR: 4,270,000–4,995,000)	4,490,000 (IQR: 4,205,000–4,900,000)	4,620,000 (IQR: 4,300,000–4,860,000)	4,630,000 (IQR: 4,280,000–5,067,500)	
Hemoglobin (g/dL)					11.7–15.5
Mean ± SD	13.28 ± 1.86	12.96 ± 1.91	13.2 ± 2.06	13.41 ± 1.77	
CRP (mg/L)					0–5
Median (IQR: Q1–Q3)	84.72 (IQR: 26.29–140.39)	77.15 (IQR: 28.54–135.85)	74.24 (IQR: 9.17–149.86)	92.09 (IQR: 29.04–141.78)	
Serum sodium (mmol/L)					135–148
Median (IQR: Q1–Q3)	141.6 (IQR: 139.2–144)	142.1 (IQR: 139.6–144.47)	142.9 (IQR: 140.2–144.9)	141.3 (IQR: 138.8–143.6)	
Serum potassium (mmol/L)					3.7–5.3
Mean ± SD	4.01 ± 0.50	4.01 ± 0.60	4.02 ± 0.52	4.01 ± 0.46	
Serum chloride (mmol/L)					98–109
Median (IQR: Q1–Q3)	101.14 ± 3.24	101.3 (IQR: 99.3–103.52)	101.8 (IQR: 100.7–104.4)	101 (IQR: 99–103)	

* WBC = white blood cell, RBC = red blood cell.

**Table 3:** Yearly variations in *Salmonella* serogroups.

Serogroup	2018	2019	2020	2021	2022	2023	2024	Total
B (n, %)	45 (17%)	57 (36.8%)	5 (45.5%)	9 (32.1%)	8 (16%)	13 (18.8%)	15 (12.4%)	152 (21.8%)
C (n, %)	29 (11%)	29 (18.7%)	2 (18.2%)	5 (17.9%)	13 (26%)	9 (13%)	11 (9.1%)	98 (14%)
D (n, %)	188 (71.2%)	67 (43.2%)	4 (36.4%)	14 (50%)	29 (58%)	47 (68.1%)	95 (78.5%)	444 (63.6%)
E (n, %)	2 (0.8%)	0 (0%)	0 (0%)	0 (0%)	0 (0%)	0 (0%)	0 (0%)	2 (0.3%)
G (n, %)	0 (0%)	1 (0.6%)	0 (0%)	0 (0%)	0 (0%)	0 (0%)	0 (0%)	1 (0.1)
UN (n, %) *	0(0%)	1 (0.6%)	0 (0%)	0 (0%)	0 (0%)	0 (0%)	0 (0%)	1 (0.1%)

* UN = unknown.

## Data Availability

The original contributions presented in this study are included in the article/Supplementary Materials. Further inquiries can be directed to the corresponding author.

## Supplementary Materials

The following supporting information can be downloaded at: https://www.mdpi.com/article/10.3390/microorganisms13122655/s1, Table S1: Yearly variations in patient demographics, length of hospital stay and comorbidities; Table S2: Overview of patients diagnosed with iNTS.

## Abbreviations

The following abbreviations are used in this manuscript:

NTS	Non-typhoidal *Salmonella*
iNTS	Invasive non-typhoidal *Salmonella*
WBC	White blood cell
RBC	Red blood cell
CRP	C-Reactive Protein
AMP	Ampicillin
SXT	Trimethoprim-sulfamethoxazole
CIP	Ciprofloxacin
MDR	Multidrug resistance
EFSA	European Food Safety Authority
ECDC	European Centre for Disease Prevention and Control

## References

[1] Antony B. (2022). Non Typhoidal Salmonellae and Its Aetiological Spectrum—An Overview with Indian Perspective. IP Int. J. Med. Microbiol. Trop. Dis..

[2] Mulla Z.D. (2004). Re: “Epidemiology of Salmonellosis in California, 1990–1999: Morbidity, Mortality, and Hospitalization Costs”. Am. J. Epidemiol..

[3] Sanni A.O., Onyango J., Rota A.F., Mikecz O., Usman A., PicaCiamarra U., Fasina F.O. (2023). Underestimated Economic and Social Burdens of Non-Typhoidal Salmonella Infections: The One Health Perspective from Nigeria. One Health.

[4] Kim S., Kang H., Excler J.-L., Kim J.H., Lee J.-S. (2024). The Economic Burden of Non-Typhoidal Salmonella and Invasive Non-Typhoidal Salmonella Infection: A Systematic Literature Review. Vaccines.

[5] Nemhauser J.B., Centers for Disease Control (U.S.) (2023). CDC Yellow Book 2024: Health Information for International Travel.

[6] European Food Safety Authority (EFSA), European Centre for Disease Prevention and Control (ECDC) (2024). The European Union Summary Report on Antimicrobial Resistance in Zoonotic and Indicator Bacteria from Humans, Animals and Food in 2021–2022. EFSA J..

[7] European Food Safety Authority, European Centre for Disease Prevention and Control (2022). The European Union One Health 2021 Zoonoses Report. EFSA J..

[8] Havelaar A.H., Ivarsson S., Löfdahl M., Nauta M.J. (2013). Estimating the True Incidence of Campylobacteriosis and Salmonellosis in the European Union, 2009. Epidemiol. Infect..

[9] O’Boyle H., Kirpalani A., Weiss L., Hames N., Li R., Leong T., Gonzalez M., Shane A.L., Charvat C. (2022). Management and Outcomes of Salmonella Gastroenteritis in the Era of Rapid Molecular Testing. Hosp. Pediatr..

[10] Galán-Relaño Á., Valero Díaz A., Huerta Lorenzo B., Gómez-Gascón L., Mena Rodríguez M.Á., Carrasco Jiménez E., Pérez Rodríguez F., Astorga Márquez R.J. (2023). Salmonella and Salmonellosis: An Update on Public Health Implications and Control Strategies. Animals.

[11] Dudhane R.A., Bankar N.J., Shelke Y.P., Badge A.K. (2023). The Rise of Non-Typhoidal Salmonella Infections in India: Causes, Symptoms, and Prevention. Cureus.

[12] Ehuwa O., Jaiswal A.K., Jaiswal S. (2021). Salmonella, Food Safety and Food Handling Practices. Foods.

[13] Uzairue L.I., Shittu O.B., Ojo O.E., Obuotor T.M., Olanipekun G., Ajose T., Arogbonlo R., Medugu N., Ebruke B., Obaro S.K. (2023). Antimicrobial Resistance and Virulence Genes of Invasive *Salmonella enterica* from Children with Bacteremia in North-Central Nigeria. SAGE Open Med..

[14] Abebe E., Gugsa G., Ahmed M. (2020). Review on Major Food-Borne Zoonotic Bacterial Pathogens. J. Trop. Med..

[15] Chen H., Qiu H., Zhong H., Cheng F., Wu Z., Shi T. (2023). Non-Typhoidal Salmonella Infections Among Children in Fuzhou, Fujian, China: A 10-Year Retrospective Review from 2012 to 2021. Infect. Drug Resist..

[16] Stanaway J.D., Parisi A., Sarkar K., Blacker B.F., Reiner R.C., Hay S.I., Nixon M.R., Dolecek C., James S.L., Mokdad A.H. (2019). The Global Burden of Non-Typhoidal Salmonella Invasive Disease: A Systematic Analysis for the Global Burden of Disease Study 2017. Lancet Infect. Dis..

[17] Marchello C.S., Birkhold M., Crump J.A., Martin L.B., Ansah M.O., Breghi G., Canals R., Fiorino F., Gordon M.A., Kim J.-H. (2022). Complications and Mortality of Non-Typhoidal Salmonella Invasive Disease: A Global Systematic Review and Meta-Analysis. Lancet Infect. Dis..

[18] Uche I.V., MacLennan C.A., Saul A. (2017). A Systematic Review of the Incidence, Risk Factors and Case Fatality Rates of Invasive Nontyphoidal Salmonella (iNTS) Disease in Africa (1966 to 2014). PLoS Negl. Trop. Dis..

[19] Morpeth S.C., Ramadhani H.O., Crump J.A. (2009). Invasive Non-Typhi *Salmonella* Disease in Africa. Clin. Infect. Dis..

[20] Zha L., Garrett S., Sun J. (2019). Salmonella Infection in Chronic Inflammation and Gastrointestinal Cancer. Diseases.

[21] Van Elsland D.M., Duijster J.W., Zhang J., Stévenin V., Zhang Y., Zha L., Xia Y., Franz E., Sun J., Mughini-Gras L. (2022). Repetitive Non-Typhoidal Salmonella Exposure Is an Environmental Risk Factor for Colon Cancer and Tumor Growth. Cell Rep. Med..

[22] Lamichhane B., Mawad A.M.M., Saleh M., Kelley W.G., Harrington P.J., Lovestad C.W., Amezcua J., Sarhan M.M., El Zowalaty M.E., Ramadan H. (2024). Salmonellosis: An Overview of Epidemiology, Pathogenesis, and Innovative Approaches to Mitigate the Antimicrobial Resistant Infections. Antibiotics.

[23] Eikmeier D., Medus C., Smith K. (2018). Incubation Period for Outbreak-Associated, Non-Typhoidal Salmonellosis Cases, Minnesota, 2000–2015. Epidemiol. Infect..

[24] Motladiile T.W., Tumbo J.M., Malumba A., Adeoti B., Masekwane N.J., Mokate O.M.R., Sebekedi O.C. (2019). Salmonella Food-Poisoning Outbreak Linked to the National School Nutrition Programme, North West Province, South Africa. S. Afr. J. Infect. Dis..

[25] Yang X., Jin K., Yang F., Yuan G., Liu W., Xiang L., Wu Z., Li Z., Mao J., Shen J. (2017). Nontyphoidal Salmonella Gastroenteritis in Baoshan, Shanghai, China, 2010 to 2014: An Etiological Surveillance and Case-Control Study. J. Food Prot..

[26] Sevilla-Navarro S., Catalá-Gregori P., García C., Cortés V., Marin C. (2020). Salmonella Infantis and Salmonella Enteritidis Specific Bacteriophages Isolated Form Poultry Faeces as a Complementary Tool for Cleaning and Disinfection against Salmonella. Comp. Immunol. Microbiol. Infect. Dis..

[27] Chang Y.-J., Chen Y.-C., Chen N.-W., Hsu Y.-J., Chu H.-H., Chen C.-L., Chiu C.-H. (2021). Changing Antimicrobial Resistance and Epidemiology of Non-Typhoidal Salmonella Infection in Taiwanese Children. Front. Microbiol..

[28] Hengkrawit K., Tangjade C. (2022). Prevalence and Trends in Antimicrobial Susceptibility Patterns of Multi-Drug-Resistance Non-Typhoidal Salmonella in Central Thailand, 2012–2019. Infect. Drug Resist..

[29] Sarkodie-Addo P., Aglomasa B.C., Donkor E.S. (2025). Prevalence and Antimicrobial Resistance Patterns of Nontyphoidal Salmonella in Ghana: A Systematic Review and Meta-Analysis. Trop. Med. Health.

[30] Li W., Han H., Liu J., Ke B., Zhan L., Yang X., Tan D., Yu B., Huo X., Ma X. (2023). Antimicrobial Resistance Profiles of Salmonella Isolates from Human Diarrhea Cases in China: An Eight-Year Surveilance Study. One Health Adv..

[31] Gong B., Li H., Feng Y., Zeng S., Zhuo Z., Luo J., Chen X., Li X. (2022). Prevalence, Serotype Distribution and Antimicrobial Resistance of Non-Typhoidal Salmonella in Hospitalized Patients in Conghua District of Guangzhou, China. Front. Cell. Infect. Microbiol..

[32] Crump J.A., Nyirenda T.S., Kalonji L.M., Phoba M.-F., Tack B., Platts-Mills J.A., Gordon M.A., Kariuki S.M. (2023). Nontyphoidal *Salmonella* Invasive Disease: Challenges and Solutions. Open Forum Infect. Dis..

[33] Tăbăran A., Dan S.D., Colobaţiu L.M., Mihaiu M., Condor S., Mărgăoan R., Crişan-Reget O.L. (2024). Evaluation of Multidrug Resistance of Salmonella Isolated from Pork Meat Obtained from Traditional Slaughter Systems in Romania. Microorganisms.

[34] Forgaciu A., Tabaran A., Colobatiu L., Mihaiu R., Dan S.D., Mihaiu M. (2022). Concerning Increase in Antimicrobial Resistance Patterns of Pathogenic Strains of Salmonella Isolated in Poultry Meat Products. Antibiotics.

[35] Wang T., Li W., Zhang R., Wen J., Liu S., Jiang Y., Lin L., Chen W., Liang J., Ma X. (2025). Epidemiological Features of Nontyphoidal Salmonella Infections Reported to Foodborne Disease Surveillance System in China, 2013–2022. BMC Public Health.

[36] Assefa A., Girma M. (2019). Prevalence and Antimicrobial Susceptibility Patterns of Salmonella and Shigella Isolates among Children Aged below Five Years with Diarrhea Attending Robe General Hospital and Goba Referral Hospital, South East Ethiopia. Trop. Dis. Travel Med. Vaccines.

[37] Kim C., Frost I., Naylor N.R., Au H., Kim Y., Lee Y., Bzymek A., Majgier K., Moldoveanu A.L., Salman O.M. (2025). Length of Hospital Stay and Associated Treatment Costs for Patients with Susceptible and Antibiotic-Resistant *Salmonella* Infections: A Systematic Review and Meta-Analysis. BMJ Open.

[38] Gil Prieto R., Alejandre C.G., Meca A.Á., Barrera V.H., De Miguel Á.G. (2009). Epidemiology of Hospital-Treated Salmonella Infection; Data from a National Cohort over a Ten-Year Period. J. Infect..

[39] Hagedoorn N.N., Murthy S., Birkhold M., Marchello C.S., Crump J.A., The Vacc-iNTS Consortium Collaborators (2024). Prevalence and Distribution of Non-Typhoidal *Salmonella Enterica* Serogroups and Serovars Isolated from Normally Sterile Sites: A Global Systematic Review. Epidemiol. Infect..

[40] Kumar G., Kumar S., Jangid H., Dutta J., Shidiki A. (2025). The Rise of Non-Typhoidal Salmonella: An Emerging Global Public Health Concern. Front. Microbiol..

[41] Bloomfield S.J., Janecko N., Palau R., Alikhan N.-F., Mather A.E. (2023). Genomic Diversity and Epidemiological Significance of Non-Typhoidal Salmonella Found in Retail Food Collected in Norfolk, UK. Microb. Genomics.

[42] Mkangara M. (2023). Prevention and Control of Human Salmonella Enterica Infections: An Implication in Food Safety. Int. J. Food Sci..

[43] European Food Safety Authority and European Centre for Disease Prevention and Control (EFSA and ECDC) (2018). The European Union Summary Report on Trends and Sources of Zoonoses, Zoonotic Agents and Food-borne Outbreaks in 2017. EFSA J..

[44] Alvarez D.M., Barrón-Montenegro R., Conejeros J., Rivera D., Undurraga E.A., Moreno-Switt A.I. (2023). A Review of the Global Emergence of Multidrug-Resistant Salmonella Enterica Subsp. Enterica Serovar Infantis. Int. J. Food Microbiol..

[45] Yoon K.-B., Song B.-J., Shin M.-Y., Lim H.-C., Yoon Y.-H., Jeon D.-Y., Ha H., Yang S.-I., Kim J.-B. (2017). Antibiotic Resistance Patterns and Serotypes of *Salmonella* Spp. Isolated at Jeollanam-Do in Korea. Osong Public Health Res. Perspect..

[46] Herbinger K.-H., Hanus I., Schunk M., Beissner M., Von Sonnenburg F., Löscher T., Bretzel G., Hoelscher M., Nothdurft H.D., Huber K.L. (2016). Elevated Values of C-Reactive Protein Induced by Imported Infectious Diseases: A Controlled Cross-Sectional Study of 11,079 Diseased German Travelers Returning from the Tropics and Subtropics. Am. Soc. Trop. Med. Hyg..

[47] Yang Y.-J., Huang M.-C., Wang S.-M., Wu J.-J., Cheng C.-P., Liu C.-C. (2002). Analysis of Risk Factors for Bacteremia in Children with Nontyphoidal Salmonella Gastroenteritis. Eur. J. Clin. Microbiol. Infect. Dis..

[48] Patel S., McCormick B.A. (2014). Mucosal Inflammatory Response to Salmonella Typhimurium Infection. Front. Immunol..

[49] Splichal I., Rychlik I., Splichalova I., Karasova D., Splichalova A. (2020). Toll-Like Receptor 4 Signaling in the Ileum and Colon of Gnotobiotic Piglets Infected with Salmonella Typhimurium or Its Isogenic ∆rfa Mutants. Toxins.

[50] Jones T.F., Ingram L.A., Cieslak P.R., Vugia D.J., Tobin-D’Angelo M., Hurd S., Medus C., Cronquist A., Angulo F.J. (2008). Salmonellosis Outcomes Differ Substantially by Serotype. J. Infect. Dis..

[51] Medalla F., Gu W., Friedman C.R., Judd M., Folster J., Griffin P.M., Hoekstra R.M. (2021). Increased Incidence of Antimicrobial-Resistant Nontyphoidal *Salmonella* Infections, United States, 2004–2016. Emerg. Infect. Dis..

[52] Buzilă E.R., Gatej R., Trifan C., Vremera T., Leustean M., David A., Bosogea D.C., Barbu G., Gatea A., Ilie C. (2025). Genetic Characterization of Salmonella and Analysis of Ciprofloxacin Resistance Using Sanger Technique in Romania, 2024. Bacteria.

[53] Cao G., Zhao S., Kuang D., Hsu C.-H., Yin L., Luo Y., Chen Z., Xu X., Strain E., McDermott P. (2023). Geography Shapes the Genomics and Antimicrobial Resistance of Salmonella Enterica Serovar Enteritidis Isolated from Humans. Sci. Rep..

[54] Marino A., Maniaci A., Lentini M., Ronsivalle S., Nunnari G., Cocuzza S., Parisi F.M., Cacopardo B., Lavalle S., La Via L. (2025). The Global Burden of Multidrug-Resistant Bacteria. Epidemiologia.

[55] Marino A., Augello E., Bellanca C.M., Cosentino F., Stracquadanio S., La Via L., Maniaci A., Spampinato S., Fadda P., Cantarella G. (2025). Antibiotic Therapy Duration for Multidrug-Resistant Gram-Negative Bacterial Infections: An Evidence-Based Review. Int. J. Mol. Sci..

[56] Mihaiu L., Lapusan A., Tanasuica R., Sobolu R., Mihaiu R., Oniga O., Mihaiu M. (2014). First Study of Salmonella in Meat in Romania. J. Infect. Dev. Ctries..

[57] Balea L.B., Glasdam S. (2024). Practices, Strategies, and Challenges in Antibiotic Treatment and Prevention of Antimicrobial Resistance from the Perspectives of Romanian Community Pharmacists and General Practitioners: A Goffman-Inspired Qualitative Interview Study. Front. Antibiot..

[58] Turgeon P., Murray R., Nesbitt A. (2017). Hospitalizations Associated with Salmonellosis among Seniors in Canada, 2000–2010. Epidemiol. Infect..

[59] Lin R.J., Evans A.T., Chused A.E., Unterbrink M.E. (2013). Anemia in General Medical Inpatients Prolongs Length of Stay and Increases 30-Day Unplanned Readmission Rate. South. Med. J..

[60] Katiyo S., Muller-Pebody B., Minaji M., Powell D., Johnson A.P., De Pinna E., Day M., Harris R., Godbole G. (2019). Epidemiology and Outcomes of Nontyphoidal *Salmonella* Bacteremias from England, 2004 to 2015. J. Clin. Microbiol..

[61] Cummings P.L., Kuo T., Javanbakht M., Shafir S., Wang M., Sorvillo F. (2016). Salmonellosis Hospitalizations in the United States: Associated Chronic Conditions, Costs, and Hospital Outcomes, 2011, Trends 2000–2011. Foodborne Pathog. Dis..

[62] Pagani G., Parenti M., Franzetti M., Pezzati L., Bassani F., Osnaghi B., Vismara L., Pavia C., Mirri P., Rusconi S. (2023). Invasive and Non-Invasive Human Salmonellosis Cases Admitted between 2015 and 2021 in Four Suburban Hospitals in the Metropolitan Area of Milan (Italy): A Multi-Center Retrospective Study. Pathogens.

[63] Mughini-Gras L., Pijnacker R., Duijster J., Heck M., Wit B., Veldman K., Franz E. (2020). Changing Epidemiology of Invasive Non-Typhoid Salmonella Infection: A Nationwide Population-Based Registry Study. Clin. Microbiol. Infect..

[64] Sia S., Ablola F., Lagrada M., Olorosa A., Gayeta J., Limas M., Jamoralin M., Macaranas P.K., Espiritu H.G., Borlaza J.J. (2023). Epidemiology and Antimicrobial Resistance Profile of Invasive Non-Typhoidal Salmonella from the Philippines Antimicrobial Resistance Surveillance Program, 2014–2018. West. Pac. Surveill. Response J..

[65] Lunguya O., Lejon V., Phoba M.-F., Bertrand S., Vanhoof R., Glupczynski Y., Verhaegen J., Muyembe-Tamfum J.-J., Jacobs J. (2013). Antimicrobial Resistance in Invasive Non-Typhoid Salmonella from the Democratic Republic of the Congo: Emergence of Decreased Fluoroquinolone Susceptibility and Extended-Spectrum Beta Lactamases. PLoS Negl. Trop. Dis..

[66] Song W., Shan Q., Qiu Y., Lin X., Zhu C., Zhuo Z., Wang C., Tong J., Li R., Wan C. (2022). Clinical Profiles and Antimicrobial Resistance Patterns of Invasive Salmonella Infections in Children in China. Eur. J. Clin. Microbiol. Infect. Dis..

[67] Israel Y., Muhsen K., Rokney A., Adler A. (2022). Epidemiological and Clinical Characteristics of Non-Typhoidal Salmonella Bloodstream Infections in Central Israel: A Case-Control Study. Microorganisms.

[68] Björklund L., Mattisson Y., Bläckberg A., Sunnerhagen T., Ljungquist O. (2024). A Population-Based Study on the Incidence, Risk Factors, and Outcome of Salmonella Bloodstream Infections in South Sweden 2012–2022. Infect. Dis. Ther..

[69] Haselbeck A.H., Panzner U., Im J., Baker S., Meyer C.G., Marks F. (2017). Current Perspectives on Invasive Nontyphoidal Salmonella Disease. Curr. Opin. Infect. Dis..

[70] Li C.-W., Chen P.-L., Lee N.-Y., Lee H.-C., Chang C.-M., Lee C.-C., Ko W.-C. (2012). Non-Typhoidal Salmonella Bacteremia among Adults: An Adverse Prognosis in Patients with Malignancy. J. Microbiol. Immunol. Infect..

[71] Mori N., Szvalb A.D., Adachi J.A., Tarrand J.J., Mulanovich V.E. (2021). Clinical Presentation and Outcomes of Non-Typhoidal Salmonella Infections in Patients with Cancer. BMC Infect. Dis..

[72] Hsu R.-B., Chen R.J., Lin F.-Y., Chu S.-H. (2005). Influence of Ciprofloxacin Resistance on Risk Factors for Endovascular Infection in Patients with Infection Due to Group C Nontyphoid Salmonellae. Clin. Infect. Dis..

[73] Wetchasirigul S., Puangseree J., Angkititrakul S., Prathan R., Srisanga S., Chuanchuen R. (2025). Antimicrobial Resistance and Plasmid-Associated Virulence Genes in *Salmonella* Isolated from Pigs, Pork, and Humans in Border Provinces of Thailand and Neighboring Countries. PeerJ.

[74] Khajanchi B.K., Foley S.L. (2022). Antimicrobial Resistance and Increased Virulence of Salmonella. Microorganisms.

