# Supplementary material for: Seven Years of Salmonella: Changing Resistance and Clinical Insights
Source: Microorganisms. 2025 Nov 22;13(12):2655. doi: 10.3390/microorganisms13122655 (PMC12734770; doi:10.3390/microorganisms13122655)
Supplement: Supplementary file 1 [file microorganisms-13-02655-s001.zip › microorganisms-3983271-supplementary.pdf]

Table S1. Yearly variations of patient demographics, length of hospital stay and comorbidities.

Parameter	2018	2019	2020	2021	2022	2023	2024
Number of patients (n)	264	155	11	28	50	69	121
Sex (n, %)							
Male	143 (54.2%)	82 (52.9%)	7 (63.6%)	15 (53.6%)	30 (60%)	30 (43.5%)	60 (49.6%)
Female	121 (45.8%)	73 (47.1%)	4 (36.4%)	13 (46.4%)	20 (40%)	39 (56.5%)	61 (50.4%)
Residence (n, %)							
Urban	104 (39.4%)	63 (40.6%)	4 (36.4%)	12 (42.9%)	28 (56%)	33 (47.8%)	45 (37.2%)
Rural	160 (60.6%)	92 (59.4%)	7 (63.6%)	16 (57.1%)	22 (44%)	36 (52.2%)	76 (62.8%)
Age (years)							
Median (IQR: Q1-Q3)	18 (IQR: 7-35)	28 (IQR: 7-57)	31 (IQR: 26-75)	34 (IQR: 20-51.75)	34.5 (IQR: 20.5-52.25)	31 (IQR: 18-62)	34 (IQR: 15-56)
<18 years (n, %)	128 (48.5%)	60 (38.7%)	2 (18.2%)	5 (17.9%)	10 (20%)	16 (23.2%)	31 (25.6%)
≥18 years (n, %)	136 (51.5%)	95 (61.3%)	9 (81.8%)	23 (82.1%)	40 (80%)	53 (76.8%)	90 (74.4%)
Length of hospital stay (days) Median (IQR: Q1-Q3)	5 (IQR: 3-6)	5 (IQR: 3-7)	5 (IQR: 1-9)	5 (IQR: 1.25-7)	5 (IQR: 3-6)	4 (IQR: 3-7)	5 (IQR: 3-6)
Comorbidities (n, %)							
Diabetes mellitus	9 (3.4%)	9 (5.8%)	0 (0%)	4 (14.3%)	4 (8%)	6 (8.7%)	8 (6.6%)
Cardiovascular disease	23 (8.7%)	29 (18.7)	2 (18.2%)	7 (25%)	10 (20%)	16 (23.2%)	30 (24.8%)
Pulmonary disease	5 (1.9%)	7 (4.5%)	0 (0%)	1 (3.6%)	1 (2%)	2 (2.9%)	5 (4.1%)
Malignancy (all types)	0 (0%)	5 (3.2%)	0 (0%)	0 (0%)	1 (2%)	3 (4.3%)	6 (5%)

Table S2. Overview of patients diagnosed with iNTS.

Parameter	Value
Number of cases (n, %)	14 (2%)
Sex (n, %)	
Male	7 (50%)
Female	7 (50%)
Age (years) Median (IQR: Q1-Q3)	71.5 (IQR: 58.5-76.25)
Hospitalisation (days) Median (IQR: Q1-Q3)	12 (IQR: 8.5-22.75)
Residence (n, %)	
Urban	5 (35.7%)
Rural	9 (64.3%)
Biological Parameters (mean ± SD)	
WBC* (cells/mm^3)	7470.71 ± 2887.82
Neutrophils (cells/mm^3)	5806.43 ± 2771.11
RBC* (cells/mm^3)	4,077,857.14 ± 664,775.01
Hemoglobin (g/dL)	12.42 ± 2.02
CRP (mg/L)	82.82 ± 69.51
Serum sodium (mmol/L)	141.40 ± 4.34
Serum potassium (mmol/L)	3.78 ± 0.45
Serum chloride (mmol/L)	101.65 ± 4.09
Comorbidities (n, %)	
Diabetes mellitus	3 (21.4%)
Cardiovascular disease	6 (42.9%)
Pulmonary disease	1 (7.1%)
Malignancy (all types)	2 (14.3%)
Serogroup (n, %)	
B	3 (21.4%)
C	1 (7.1%)
D	9 (64.3%)
UN *	1 (7.1%)
Antibiotic Resistance (n, %)	
AMP	0 (0%)
SXT	0 (0%)
CIP	5 (35.7%)
MDR	0 (0%)

* UN=unknown; WBC=white blood cell; RBC=red blood cell.
